# Solitary Mucosal Neuroma of the Hard Palate Without Multiple Endocrine Neoplasia Type 2B: A Rare Non-syndromic Case Report and Literature Review

**DOI:** 10.7759/cureus.105626

**Published:** 2026-03-21

**Authors:** Haris KY, Sunita Gupta, Pavithra Ramasamy, Sujoy Ghosh, Aadithya B Urs

**Affiliations:** 1 Oral Medicine and Radiology, Maulana Azad Institute of Dental Sciences, New Delhi, IND; 2 Oral Pathology and Microbiology, Maulana Azad Institute of Dental Sciences, New Delhi, IND

**Keywords:** hard palate, immunohistochemistry, men 2b, mucosal neuroma, multiple endocrine neoplasia type 2b, neuroma, non-syndromic, ret proto-oncogene, s-100, solitary neuroma

## Abstract

Mucosal neuromas are benign proliferations of peripheral nerves that most commonly occur as part of multiple endocrine neoplasia type 2B (MEN2B), a hereditary syndrome associated with germline mutations of the RET proto-oncogene. MEN2B is characterized by mucosal neuromas, medullary thyroid carcinoma, pheochromocytoma, intestinal ganglioneuromatosis, and marfanoid habitus. Oral mucosal neuromas often represent the earliest manifestation of the syndrome and therefore have important diagnostic significance. However, isolated mucosal neuromas occurring in the absence of MEN2B are extremely rare, particularly within the oral cavity. This case report describes a 25-year-old female who presented with a solitary, firm, painless mass on her right posterior hard palate. Radiographic examination revealed no osseous involvement. Following an excisional biopsy, microscopic examination revealed unencapsulated proliferation of tortuous and haphazardly arranged neural bundles within a collagenous stroma. Immunohistochemical analysis demonstrated strong and diffuse positivity for S-100 protein, confirming the diagnosis of mucosal neuroma. Comprehensive systemic evaluation, including endocrine assessment, biochemical testing, and thyroid ultrasonography, revealed no evidence of MEN2B. The patient demonstrated satisfactory postoperative healing with no recurrence during eight months of follow-up. Thus, this case highlights an exceedingly rare presentation of a solitary mucosal neuroma at an unusual site occurring in the absence of MEN2B. While mucosal neuromas are typically pathognomonic for MEN2B, solitary forms are rare, particularly when located on the hard palate. This report adds to the limited literature on non-syndromic oral mucosal neuromas and emphasizes the importance of clinicopathologic correlation and long-term surveillance.

## Introduction

Mucosal neuromas are benign neural proliferations characterized by hyperplasia of peripheral nerve bundles within the mucosal connective tissue. These lesions are classically associated with multiple endocrine neoplasia type 2B (MEN2B), an autosomal dominant disorder caused by activating mutations in the RET proto-oncogene, resulting in abnormal neural tissue proliferation [1,2]. MEN2B is characterized by mucosal neuromas, medullary thyroid carcinoma, pheochromocytoma, intestinal ganglioneuromatosis, and distinctive skeletal features including marfanoid habitus [2-4].

Oral mucosal neuromas are frequently among the earliest clinical manifestations of MEN2B and often involve the lips, tongue, and buccal mucosa. In many patients, these lesions precede the diagnosis of medullary thyroid carcinoma by several years, making their recognition critical for early identification of the syndrome [3,4]. Early detection of MEN2B allows prophylactic thyroidectomy, which significantly improves patient survival and prognosis. In contrast, isolated mucosal neuromas not associated with MEN2B are exceedingly rare. These lesions occur without systemic abnormalities or identifiable RET mutations and are typically solitary. The pathogenesis of such lesions remains unclear, although localized neural hyperplasia or reactive proliferation has been proposed [5].

Among reported cases of isolated mucosal neuromas within the oral cavity, the hard palate is an exceptionally uncommon location, with very few cases documented in the literature [6]. While mucosal neuromas are well-recognized features of MEN2B, solitary neuromas occurring in the absence of systemic disease are rare, with involvement of the hard palate being particularly uncommon. Because of their rarity and nonspecific clinical appearance, these lesions may be mistaken for other benign oral soft tissue tumors such as fibroma, neurofibroma, or minor salivary gland neoplasms. The present report describes a case of solitary mucosal neuroma arising on the posterior hard palate in a young female without evidence of MEN2B, along with a review of previously reported cases.

## Case presentation

A 25-year-old female presented to the Department of Oral Medicine and Radiology at a tertiary dental care center with a chief complaint of a painless swelling on the right side of her palate that had been present for one month. The patient reported gradual enlargement of the lesion without associated pain, bleeding, or discharge. Her medical and family histories were unremarkable for endocrine disorders or similar lesions.

**Figure 1:**
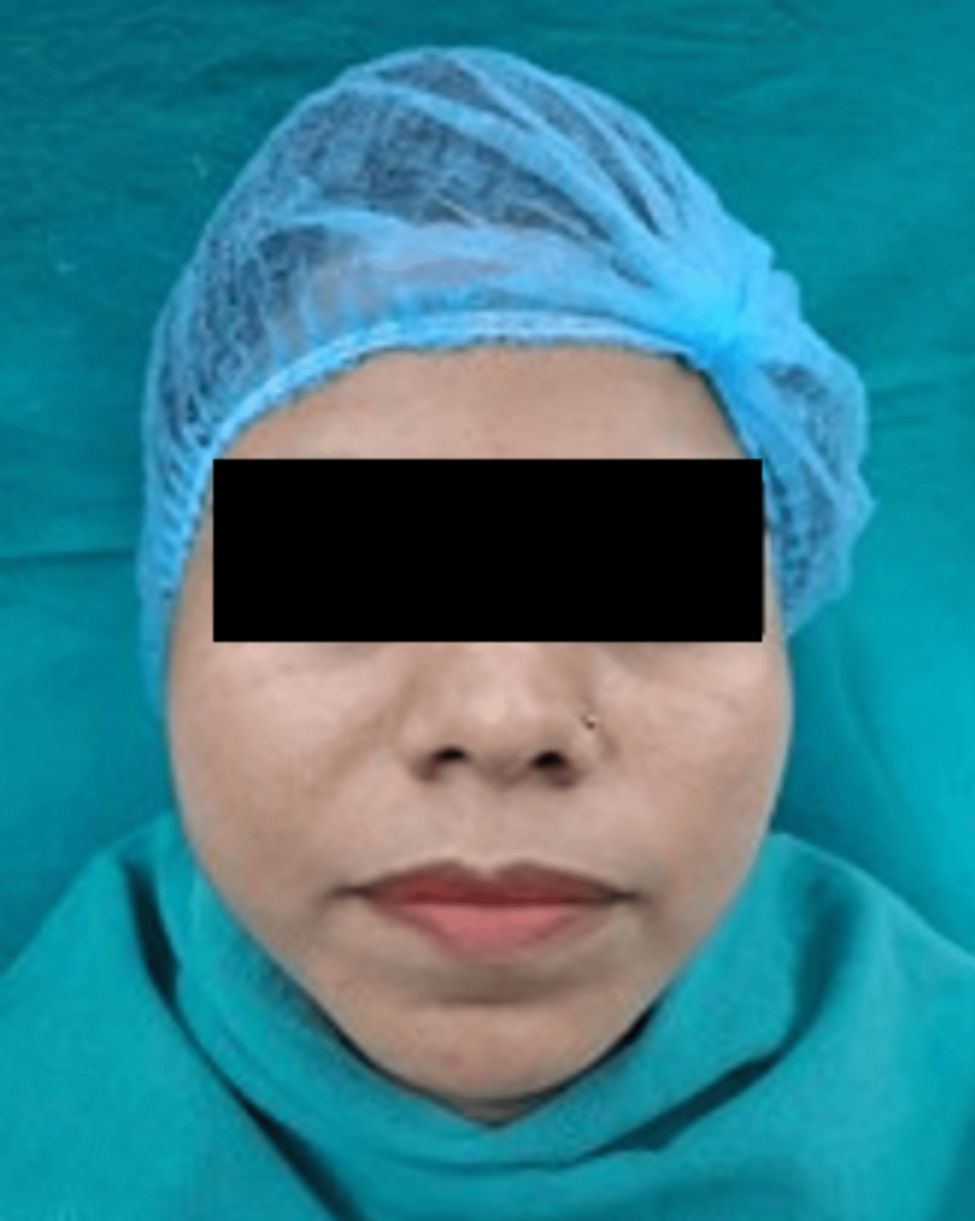
Extraoral Clinical Photograph No facial asymmetry or lip abnormalities were observed.

**Figure 2:**
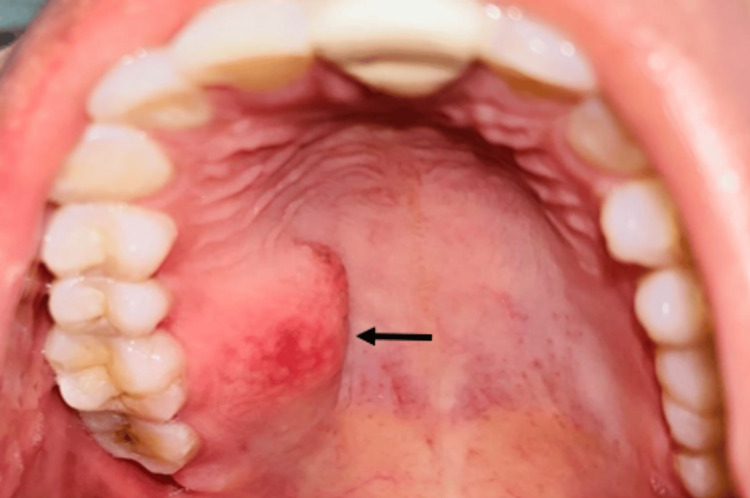
Intraoral Clinical Photograph Intraoral view showing a well-defined, dome-shaped swelling on the right posterior hard palate.

Extraoral examination showed no facial asymmetry or characteristic lip thickening (Figure 1). There were no features suggestive of marfanoid habitus. Intraoral examination revealed a solitary, dome-shaped, firm, and non-tender submucosal swelling on the right posterior hard palate in relation to teeth 15, 16, and 17 (Figure 2). The lesion measured approximately 2.5 cm in greatest dimension, had a smooth surface, and the overlying mucosa appeared intact and of normal color. The adjacent teeth were clinically healthy and vital. Based on the clinical presentation, a provisional diagnosis of a benign soft tissue lesion was considered, with differential diagnoses including fibroma, neurofibroma, traumatic neuroma, and minor salivary gland tumor.

**Figure 3:**
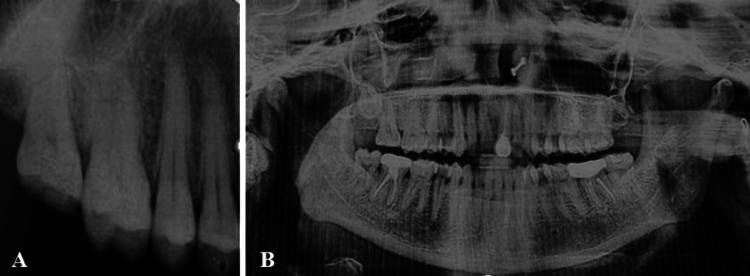
Radiographic Findings (A) Intraoral periapical radiograph showing no local osseous abnormality, except for an impacted right maxillary third molar (tooth 18). (B) Orthopantomogram demonstrating an impacted right maxillary third molar (tooth 18).

Radiographic evaluation included intraoral periapical radiograph (IOPAR) and orthopantomogram (OPG). Imaging revealed no underlying osseous involvement except for an incidental finding of an impacted maxillary right third molar (tooth 18) (Figure 3A, 3B).

**Figure 4:**
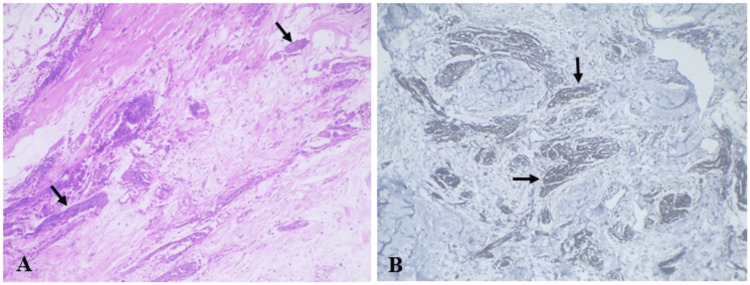
Histopathological and Immunohistochemical Findings (A) Hematoxylin and eosin (H&E) stained section showing multiple tortuous neural bundles (black arrows) composed of spindle shaped cells with wavy nuclei within a loosely collagenous stroma (×10). (B) Immunohistochemical staining showing strong S-100 positivity in the neural bundles (black arrows) (×10).

The lesion was completely excised under local anesthesia. Gross examination of the surgical specimen revealed a firm, irregular whitish-brown mass measuring approximately 2.8 × 1.9 × 0.5 cm. Microscopic examination using hematoxylin and eosin staining demonstrated interlacing bundles of spindle cells with hyperchromatic wavy nuclei arranged in multiple tortuous neural fascicles within a loosely to moderately collagenous stroma. Focal areas of collagen hyalinization were also observed (Figure 4A). Immunohistochemical analysis revealed diffuse and strong positivity for S-100 protein, confirming the neural origin of the lesion and supporting the diagnosis of mucosal neuroma (Figure 4B).

**Figure 5:**
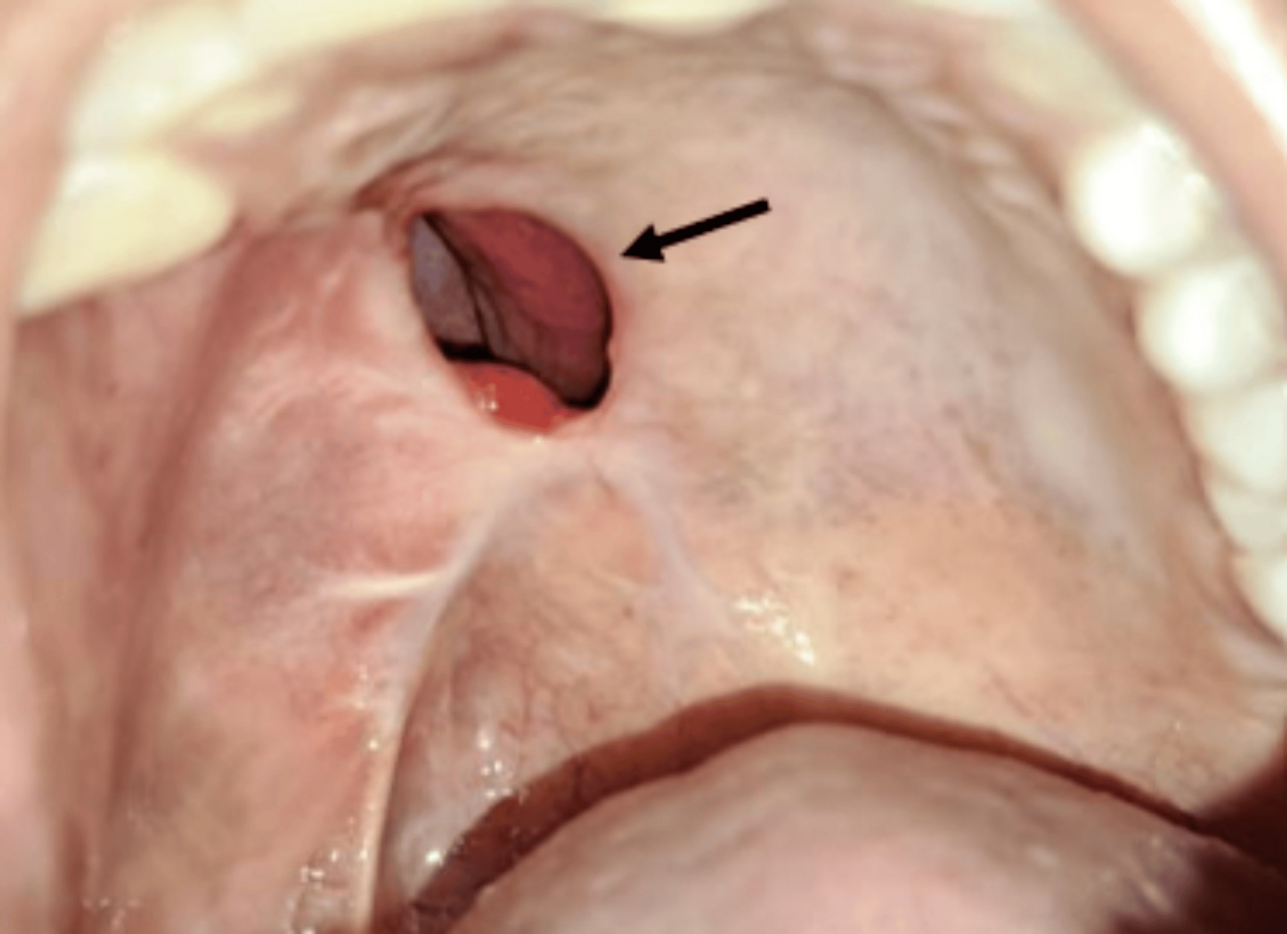
Postoperative Follow-up Follow-up at eight months showing satisfactory healing with no evidence of recurrence.

Following the histopathological diagnosis, the patient underwent systemic evaluation to exclude MEN2B. Clinical examination revealed no additional neuromas on the lips, eyelids, or tongue. The patient exhibited normal body habitus without marfanoid features. Laboratory investigations, including serum calcium, parathyroid hormone, calcitonin, and plasma metanephrines, were within normal limits. Real-time sonographic examination of the neck using a linear 5-9 MHz transducer revealed a thyroid gland with homogeneous echotexture bilaterally. The gland showed normal dimensions, with the right lobe measuring 23.3 × 24.4 × 44.1 mm (volume approximately 12.1 cc) and the left lobe measuring 22.5 × 17.5 × 46.9 mm (volume approximately 10.6 cc). No focal mass lesions or suspicious calcifications were identified in either lobe. Overall, the thyroid appeared clinically and sonographically normal, with no evidence suggestive of medullary thyroid carcinoma or suspicious cervical lymphadenopathy. Based on these findings, the lesion was diagnosed as an isolated mucosal neuroma of the hard palate without MEN2B association. The patient demonstrated satisfactory postoperative healing, and eight-month follow-up revealed no recurrence (Figure 5). She has been enrolled in a long-term surveillance program with six-monthly clinical and biochemical evaluation.

## Discussion

Mucosal neuromas represent benign proliferations of peripheral nerve tissue characterized histologically by hyperplastic nerve bundles within the mucosa. These lesions are most commonly encountered in association with MEN2B syndrome, a rare but clinically significant hereditary disorder caused by activating mutations in the RET proto-oncogene [1,2].

The RET gene encodes a transmembrane tyrosine kinase receptor involved in neural crest cell development. Activating mutations, most commonly the M918T substitution, result in constitutive activation of RET signaling pathways and abnormal proliferation of neural crest-derived cells [2]. Consequently, affected individuals develop mucosal neuromas, endocrine tumors, and characteristic phenotypic features including marfanoid habitus and thickened corneal nerves [3].

Oral mucosal neuromas are among the earliest manifestations of MEN2B and frequently involve the lips, tongue, and buccal mucosa [3]. In many patients, these lesions precede the diagnosis of medullary thyroid carcinoma by several years. Because medullary thyroid carcinoma occurs in more than 90% of MEN2B patients and may develop during childhood, early recognition of mucosal neuromas can be life-saving by prompting timely genetic testing and prophylactic thyroidectomy [4].

In contrast, isolated mucosal neuromas unassociated with MEN2B are extremely uncommon. These lesions occur without endocrine abnormalities or RET mutations and typically present as solitary nodules. The pathogenesis of these isolated lesions remains unclear. Some authors have suggested that they may represent localized hyperplastic responses of peripheral nerves rather than true neoplasms [5].

Clinically, solitary mucosal neuromas usually appear as small, smooth, dome-shaped nodules that are asymptomatic and slow growing. Because of their nonspecific appearance, they are frequently misdiagnosed clinically as fibromas, neurofibromas, traumatic neuromas, or minor salivary gland tumors [6]. In the present case, the lesion presented as a painless palatal swelling, which initially raised a broad differential diagnosis including benign connective tissue tumors and salivary gland lesions.

Histopathological examination remains the gold standard for diagnosis. Mucosal neuromas are characterized by unencapsulated, tortuous nerve bundles composed of Schwann cells and axons surrounded by perineurial cells [7]. These nerve bundles are typically arranged within a collagenous connective tissue stroma. Immunohistochemical staining demonstrates strong positivity for S-100 protein, confirming the neural origin of the lesion.

The differential diagnosis includes several neural lesions of the oral cavity. Traumatic neuroma typically develops at sites of nerve injury and exhibits disorganized nerve bundles within fibrous scar tissue. Palisaded encapsulated neuroma (PEN) shows a well-circumscribed lesion with partial capsule formation and palisading of Schwann cells [8]. Neurofibroma demonstrates a diffuse proliferation of spindle cells within a myxoid stroma and lacks the characteristic tortuous neural bundles seen in mucosal neuroma [7,8].

Because mucosal neuromas are strongly associated with MEN2B, systemic evaluation is mandatory once the diagnosis is established histologically. Recommended investigations include clinical examination for additional neuromas, biochemical assessment of calcitonin and catecholamines, and imaging of the thyroid gland [4]. Early detection of MEN2B allows prompt treatment and improves long-term outcomes. A comparative overview of the clinical, genetic, histopathological, and immunohistochemical characteristics of MEN2B-associated mucosal neuromas and isolated mucosal neuromas is presented in Table 1.

**Table 1:** Literature-Based Comparison of Clinicopathological and Immunohistochemical Features of MEN2B-Associated and Solitary Mucosal Neuromas Abbreviations: MEN2B, Multiple Endocrine Neoplasia Type 2B; MTC, Medullary Thyroid Carcinoma; EMA, Epithelial Membrane Antigen; S-100, S-100 protein

Features	MEN2B-Associated Mucosal Neuroma	Isolated/Non-syndromic Mucosal Neuroma	References
Number of lesions	Multiple and widespread	Typically solitary	5, 6, 10
Appearance	Multiple yellowish-white sessile nodules; may produce thickened or “blubbery” lips	Usually presents as a single, localized nodule	1, 5
Common sites	Lips, tongue, and multiple oral mucosal sites	Rare; reported on palate and gingiva.	5, 6, 10
Systemic association	Associated with MTC, pheochromocytoma, and marfanoid habitus	Absent	1, 3, 4
Genetics	RET proto-oncogene mutation (commonly codon 918)	No consistent genetic mutation identified	2, 3
Biochemical markers	Elevated calcitonin and catecholamine metabolites (metanephrines)	Typically within normal limits	3, 4
Histopathology	Tortuous nerve bundles with interlacing fascicles of Schwann and perineurial cells	Histologically similar; interlacing nerve bundles present	5, 8
Immunohistochemistry	Schwann cells exhibit strong S-100 positivity, while perineurial cells express EMA; axons demonstrate neurofilament positivity	Identical immunoprofile: S-100 (+), EMA (+), neurofilament (+)	5, 8
Related variants	May occur as part of MEN2B spectrum	Includes palisaded encapsulated neuroma and idiopathic mucosal neuromas	8, 9
Prognosis	Depends on associated systemic disease; high risk due to MTC	Excellent; curative with local excision	3, 4, 6, 10

The present case is notable for several reasons. First, the lesion occurred as a solitary mucosal neuroma without any systemic manifestations. Second, the hard palate is an extremely rare site for this lesion. Nishihara et al. previously reported one of the earliest documented cases of solitary mucosal neuroma arising on the hard palate [6]. Subsequent reports have also confirmed the rarity of palatal involvement.

The prognosis for isolated mucosal neuroma is excellent following complete excision. Nevertheless, long-term follow-up is recommended, as MEN2B manifestations may occasionally appear later in life. Continued surveillance, including periodic clinical examination and biochemical testing, is therefore advisable.

To date, fewer than 20 cases of isolated mucosal neuroma unassociated with MEN2B have been reported in the literature, of which only one has been documented in the hard palate, representing approximately 5% of reported oral cases (Table 2) [6]. The majority of cases occur on the lips and tongue, whereas palatal involvement remains exceedingly rare [6,9,10]. Reported patients span a wide age range with no consistent sex predilection. Surgical excision remains the treatment of choice, and recurrence is rare.

**Table 2:** Review of Reported Cases of Solitary Oral Mucosal Neuroma in the Absence of Multiple Endocrine Neoplasia Type 2B (MEN2B)

References	Country	Patients (n)	Site	Treatment	Recurrence	Follow-up
Pujol et al. [9]	Spain	1	Tongue/Lip	Excision	None	22 years
Nishihara et al. [6]	Japan	1	Hard palate	Excision	None	7 months
Qiu et al. [10]	China	1	Gingiva	Excision	None	12 months
Present case	India	1	Hard palate	Excision	None	8 months

This report has certain limitations. Genetic analysis for RET proto-oncogene mutations was not performed, which could have further supported the non-syndromic nature of the lesion. Additionally, the follow-up period of eight months is relatively short, and longer surveillance is required to definitively exclude late manifestations of MEN2B.

## Conclusions

Solitary mucosal neuroma of the hard palate is an exceptionally rare benign neural lesion that may clinically mimic other oral soft tissue tumors. Although isolated lesions have an excellent prognosis following surgical excision, recognition of mucosal neuromas should prompt careful evaluation to exclude MEN2B syndrome because of the potential association with life-threatening endocrine tumors. This case contributes to the limited literature on non-syndromic oral mucosal neuromas and highlights the importance of clinicopathological correlation, appropriate systemic evaluation, and long-term surveillance.

## References

[1] Morrison PJ, Nevin NC (1996). Multiple endocrine neoplasia type 2B (mucosal neuroma syndrome, Wagenmann-Froboese syndrome). J Med Genet.

[2] Eng C, Clayton D, Schuffenecker I (1996). The relationship between specific RET proto-oncogene mutations and disease phenotype in multiple endocrine neoplasia type 2. International RET mutation consortium analysis. JAMA.

[3] Elisei R, Matrone A, Valerio L (2019). Fifty years after the first description, MEN 2B syndrome diagnosis is still late: descriptions of two recent cases. J Clin Endocrinol Metab.

[4] Castinetti F, Waguespack SG, Machens A (2019). Natural history, treatment, and long-term follow up of patients with multiple endocrine neoplasia type 2B: an international, multicentre, retrospective study. Lancet Diabetes Endocrinol.

[5] Maymone MB, Greer RO, Burdine LK (2019). Benign oral mucosal lesions: clinical and pathological findings. J Am Acad Dermatol.

[6] Nishihara K, Yoshida H, Onizawa K, Yusa H, Fujiwara M (2004). Solitary mucosal neuroma of the hard palate: a case report. Br J Oral Maxillofac Surg.

[7] Takayama Y, Yokoo S, Yamaguchi T (2024). A case series study of solitary mucosal neuroma-rare cases of benign peripheral neurogenic tumours. Clin Pathol.

[8] Koutlas IG, Scheithauer BW (2010). Palisaded encapsulated ("solitary circumscribed") neuroma of the oral cavity: a review of 55 cases. Head Neck Pathol.

[9] Pujol RM, Matias-Guiu X, Miralles J, Colomer A, de Moragas JM (1997). Multiple idiopathic mucosal neuromas: a minor form of multiple endocrine neoplasia type 2B or a new entity?. J Am Acad Dermatol.

[10] Qiu C, Wang L, Chen H, Song Z (2021). Solitary mucosal neuroma of the gingiva without multiple endocrine neoplasia type 2B: a rare case report and literature review. Front Oral Maxillofac Med.

